# A systematic experimental evaluation of microRNA markers of human bladder cancer

**DOI:** 10.3389/fgene.2013.00247

**Published:** 2013-11-15

**Authors:** Anastasia A. Zabolotneva, Alex A. Zhavoronkov, Peter V. Shegay, Nurshat M. Gaifullin, Boris Y. Alekseev, Sergey A. Roumiantsev, Andrew V. Garazha, Olga Kovalchuk, Alexey Aravin, Anton A. Buzdin

**Affiliations:** ^1^Group for Genomic Analysis of Cell Signaling Systems, Shemyakin-Ovchinnikov Institute of Bioorganic ChemistryMoscow, Russia; ^2^Laboratory of Bioinformatics, D. Rogachyov Federal Research Center of Pediatric Hematology, Oncology and ImmunologyMoscow, Russia; ^3^First Oncology Research and Advisory CenterMoscow, Russia; ^4^Department of Oncourology, P.A. Herzen Moscow Oncological Research InstituteMoscow, Russia; ^5^Faculty of Fundamental Medicine, Lomonosov Moscow State UniversityMoscow, Russia; ^6^Laboratory of Epigenetics in Health and Disease, University of Lethbridge, LethbridgeAB, Canada; ^7^Division of Biology and Biological Engineering, California Institute of Technology, PasadenaCA, USA

**Keywords:** microRNA, bladder cancer, molecular markers, intracellular regulatory pathways, transcriptome analysis

## Abstract

**Background:** MicroRNAs (miRNAs) are a class of small RNAs that regulate gene expression. They are aberrantly expressed in many human cancers and are potential therapeutic targets and molecular biomarkers.

**Methods:** In this study, we for the first time validated the reported data on the entire set of published differential miRNAs (102 in total) through a series of transcriptome-wide experiments. We have conducted genome-wide miRNA profiling in 17 urothelial carcinoma bladder tissues and in nine normal urothelial mucosa samples using three methods: (1) An Illumina HT-12 microarray hybridization (MA) analysis (2) a suppression-subtractive hybridization (SSH) assay followed by deep sequencing (DS) and (3) DS alone.

**Results:** We show that DS data correlate with previously published information in 87% of cases, whereas MA and SSH data have far smaller correlations with the published information (6 and 9% of cases, respectively). qRT-PCR tests confirmed reliability of the DS data.

**Conclusions:** Based on our data, MA and SSH data appear to be inadequate for studying differential miRNA expression in the bladder.

**Impact:** We report the first comprehensive validated database of miRNA markers of human bladder cancer.

## Introduction

Bladder cancer (BC) is one of the most common cancers in industrially developed countries. Approximately 386,300 new BC cases and 150,200 deaths caused by BC were registered worldwide in 2008 (Jemal et al., 2008). The risk of developing BC is associated with smoking and with exposure to several other carcinogens (Kiriluk et al., 2012).

Genetic factors such as chromosomal aberrations (Hoglund, 2012), specific single nucleotide polymorphisms (SNPs) (Golka et al., 2011), mutations (Castillo-Martin et al., 2010), and epigenetic peculiarities (Kim and Kim, 2012) may contribute to tumorigenesis and the progression of BC. Some molecular features may serve as diagnostic and/or prognostic markers of tumor growth, invasiveness and metastatic potential as well as signs of disease progression (Castillo-Martin et al., 2010). Molecular diagnostics based on the detection of biomarkers in tissues or in biological fluids may be the key to detecting BC early.

At present, the most commonly used molecular markers for BC diagnostics are protein-coding genes and their products which show differential expression in tumor samples compared to normal bladder tissue (Knowles, 2008). However, non-coding small RNA molecules may be useful alternative BC biomarkers. Small non-coding RNAs take part in the regulation of major biological processes such as cell division, apoptosis, differentiation, growth, migration, etc. (Eddy, 2001; Zabolotneva et al., 2010). The best-studied non-coding RNAs are microRNAs (miRNAs). MiRNAs are 21—25-nucleotide-long RNA molecules that regulate post-transcriptional gene expression (Boyd, 2008). MiRNAs join the RNA-induced silencing complex in order to regulate a targeted messenger RNA (mRNA) through the repression of its translation and/or by guiding enzymatic cleavage of the mRNA itself (Hannon, 2002).

More than half of miRNA genes are located in cancer-associated genomic regions or in fragile chromosomal sites (Calin et al., 2004). A variety of aberrantly expressed miRNAs are also associated with different cancers (Calin et al., 2004). MiRNAs may act as oncogenes or tumor suppressors. Furthermore, different cancer types, stages, and differentiation grades may have unique miRNA expression signatures; this makes miRNAs promising biomarkers for cancer diagnosis and the prediction of tumor progression.

Expression profiles of miRNAs in cancerous and normal cells may shed light on the mechanisms of bladder carcinogenesis and can be helpful in developing BC diagnostic and prognostic assays. Previously, a comprehensive database of miRNAs associated with BC was created by performing a systematic search for published literature that reported the isolation and characterization of expression of BC-specific miRNAs (http://bladder.pparser.net/MIRMarkers.php) (Zabolotneva et al., 2012).

Overall, we identified 95 miRNAs that were differentially expressed in BC tissues and seven miRNAs that were differentially methylated in BC vs. non-cancer patients. We summarized this information in a publically available database (http://bladder.pparser.net/MIRMarkers.php) which includes miRNA names, their roles in BC, and the supporting primary literature.

However, the information about the potential diagnostic utility of these biomarkers was largely missing. Here, we evaluate a complete pool of published miRNA BC markers according to universal criteria in a series of single assays using three experimental approaches. We used Illumina HT-12 microarray hybridization (MH), suppression subtractive hybridization (SSH) followed by deep sequencing (DS), and DS alone to detect differential expression of miRNAs in BC tissues vs. normal bladder tissues. We investigated 17 human bladder urothelial carcinoma tissues and nine normal urothelial mucosa samples. The results of DS experiments were significantly closer to the literature as compared to the results of the SSH and MH assays. DS analyses allowed us to trace the expression of 38/102 individual marker miRNAs; the SSH and MH methods provided data for 42 and 33 marker miRNAs, respectively. According to our DS results, we were able to estimate, for the first time, sensitivity scores (Pencina et al., 2008) of the entire pool of bladder-marker miRNAs.

## Materials and methods

### Tissue collection and RNA isolation

Tissue samples from malignant tumors were obtained from patients operated on for BC at P.A. Hertzen Moscow Clinical Oncology Institute (Moscow, Russia) from 2009 to 2011. Normal bladder mucosae came from an autopsy of undiseased cases. Tissue samples from non-cancer controls were gathered at the same time from the Pathology Department of the Faculty of Medicine at the Moscow State University. Each sample was evaluated by a pathologist to confirm the diagnosis. All tumor samples used in this study contained >80% of tumor cells. Tissue samples were immediately placed in RNAlater (Qiagen, Germany) and then stored at −80°C. In total, 23 samples from tumors and 26 samples from normal bladder tissues were analyzed. The mean age of cancer patients at the time of resection was 62 years (range 44–82). The information on age, disease stage, and grade of the individual patients is shown on Supplementary Table S1. The mean age of healthy tissue donors was 42 years with a median of 45 (range 20–71). Frozen tissue was homogenized in Trizol (Invitrogen, USA). RNA was isolated following the manufacturer's protocol. Purified RNA was dissolved in RNase-free water and stored at −80°C. The study was approved by local ethical committees at D. Rogachyov Federal Research Center of Pediatric Hematology, Oncology and Immunology, Moscow State University, P.A. Herzen Moscow Oncological Research Institute and Shemyakin-Ovchinnikov Institute of Bioorganic chemistry.

### cDNA library generation for SSH

Two mixed RNA samples were prepared for SSH analysis. Sample 1 contained total RNA extracted from nine BC tissues. Sample 2 contained total RNA extracted from three normal bladder tissues in equal quantities. Amplified double-stranded cDNA was prepared from sample 1 and sample 2 RNAs using the Switching Mechanism at the 5′ end of the RNA Transcript (SMART) (Zhu et al., 2001). Subtractive hybridization was performed using the SSH method in both directions (sample 1 vs. sample 2 and vice versa) by Evrogen (Moscow, Russia) as described in (Diatchenko et al., 1996). The supplement contains a description of SSH and all oligonucleotide sequences (Supplementary Table S2).

For SSH libraries, we conducted one lane of 60-bp paired-end read sequencing using an Illumina GAIIx sequencer, thus generating ~45 million sets of paired end reads. Sequencing was conducted at Genoanalytica (Moscow, Russia).

### Gene expression microarray experiments

A total of 26 tissue samples, including 17 cancer and nine normal bladder mucosa specimens, were selected for microarray analysis. Total RNA was extracted using TRIzol (Life Technologies, USA) and then reverse-transcribed to cDNA and cRNA using the Ambion TotalPrep cRNA Amplification Kit (Invitrogen, USA). cRNA was quantified using a NanoDrop ND-1000 Spectrophotometer (NanoDrop Technologies, USA) and adjusted to a concentration of 150 ng/mL. Seven hundred and fifty nanogram of each library was hybridized onto the bead arrays using Illumina HumanHT-12v4 Expression BeadChip (Illumina, USA). It has >25,000 annotated human genes and >48,000 probes derived from the NCBI RefSeq (Build 36.2, Rel 22) and UniGene (Build 199) databases. The (MH) experiments were done at the University of Lethbridge, at the laboratory of Olga Kovalchuk. Full microarray datasets are available upon the request to the authors.

### Sequencing data analysis

The sequenced SSH libraries contained 25,517 and 33,329 different assembled contigs for cancer and normal tissue with a total of 102 MM and 54 MM reads, respectively. Further analysis was carried out with PostParser software (http://postparser.net) developed at the Shemyakin-Ovchinnikov Institute that allows mapping and annotating sequences of interest. For a mapping algorithm, we used the BLAST-like alignment tool (BLAT) (http://genome.ucsc.edu) with further manual fine-tuning. To distinguish target sequences, we annotated out the dataset from the National Center for Biotechnology Information (NCBI) Expressed Sequence Tags database (http://www.ncbi.nlm.nih.gov/dbEST), with intron-exon boundary information provided by the NCBI Reference Sequences project (http://www.ncbi.nlm.nih.gov/RefSeq). The database of mapped and annotated contigs is available online at (http://bladder.pparser.net.)

### Small RNA analysis

We analyzed eight cancer tissue specimens and two mixed normal tissue specimens, each of which included two or three normal tissue samples. Small RNAs from total RNA extracts were cloned as previously described (Brennecke et al., 2007; Aravin et al., 2008). Following PCR amplification, the libraries were sequenced on an Illumina GAIIx platform. The number of reads varied between the libraries, the mean value was 5.04 million reads per library. The sequencing was done in the California Institute of Technology, laboratory of Alexei Aravin. Small RNA sequencing datasets are available upon the request to the authors.

### Bioinformatic analysis of small RNA libraries

Illumina adapters were trimmed from the 3′-end of raw reads, and reads shorter than 16nt were discarded. The remaining sequences were collapsed into a non-redundant list and mapped to the reference human genome using Bowtie (version 1.1.2) (Langmead et al., 2009). Up to two mismatches were allowed. The sequences that failed to map to the genome were mapped against the artificially introduced sequences. The multiplicity count of mapped sequences was normalized to the total number of reads that mapped to the genome. All further bioinformatic analyses were done using PostParser.

### qRT-PCR assay

We used TaqMan probe-based qRT-PCR assay to assess micro RNA transcription for the same RNA samples as were used for micro RNA libraries construction and sequencing. Megaplex RT Primers, Human Pool B v. 3.0 (Applied Biosystems) were used to amplify micro RNAs, and specific TaqMan probe assays hsa-miR-100, 143, 183, 199A, 200A, 203, 205 (Life Technologies) were used to measure expression of miR100, 143, 183, 199A, 200A, 203, and 205, respectively. Each experiment was performed at least in quadruplicate.

## Results

### Experimental validation of miRNA markers with SSH

**Figure 1:**
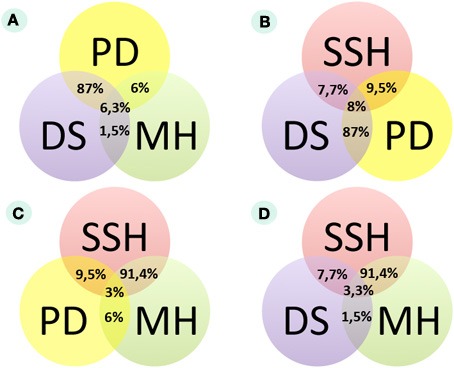
**Congruence of experimental data from three different assays according to miRNA expression in bladder cancer (MA MH, microarray hybridization analysis; SEQ DS, high-throughput deep sequencing analysis; SSH, suppressive subtractive hybridization analysis; PD, published data analysis). (A)** Comparison of published data, microarray hybridization and DS data. **(B)** Comparison of published data, SSH and DS data. **(C)** Comparison of published data, SSH and microarray hybridization data. **(D)** Comparison of DS, SSH, and microarray hybridization data.

The SSH method for transcriptome analysis is based on the selective PCR amplification of differential cDNAs (Diatchenko et al., 1996). We employed the SSH to enrich differential transcripts when comparing the two pooled RNA samples corresponding to BC and normal bladder tissues. The BC-pooled sample contained a mixture of nine BC RNA samples, while the normal sample had six pooled normal bladder RNAs. We obtained cDNA-converted subtracted libraries that were enriched for either cancer-specific (BC+) or normal-tissue-specific (BC−) cDNAs. Following DS (~21 million sets of paired-end reads for BC+ and ~23 million sets of paired-end reads for BC-libraries on the Illumina GAIIx platform), we obtained a normalized digital gene expression profile for 42/102 (41%) BC miRNA markers identified in the literature. The status of miRNA (up- or down-regulation in BC) corresponded to the published biomarker data in only four entries (9.5% of the cases) (Figure 1B). However, for two miRNAs (miR141 and miR205), contradictory data were reported [up-regulation in Billerey et al. (2001); Aravin et al. (2008); down-regulation in Adachi et al. (2011)]. According to our SSH data, miR141, miR21, and miR29C are down-regulated, and miR205 is up-regulated (online Dataset 1, http://small.mrna.ru/files/). miRNAs were considered to be differentially expressed if they met the following criteria: (1) the total normalized number of reads in both BC and normal libraries is greater or equals to 10, and (2) the average BC and normal bladder signals differ at least 1.5-times. The cancer-to-normal ratio was calculated for all differential BC signals according to the formula SR^BC^/SR^N^, where SR^BC^ and SR^N^ are normalized numbers of reads for the BC and for normal bladder samples, respectively.

### Experimental validation of miRNA markers with microarray hybridization

Our investigation of gene expression in eight clinical BC samples and four normal bladder samples using an Illumina human HT-12v4 bead array was only able to trace 33/102 (32%) individual miRNA markers that were previously reported (online Dataset 2, http://small.mrna.ru/files/). Hybridization signals for individual BC samples were compared to signals for normal tissues. Genes were considered to be differentially expressed if they met the following criteria: (1) the *p*-value < 0.01, and (2) the average BC and normal bladder signals differ at least 1.5-times. The cancer-to-normal ratio was calculated for all differential BC signals according to the formula S^BC^/Sa^N^, where S^BC^ is the BC differential hybridization signal, and Sa^N^ is the average hybridization signal for normal bladder samples.

In this test, only two micro RNAs (miR21 and miR205) showed differential expression in the BC samples under investigation, with the remaining 94% showing no differential expression. MiR205 that appeared to be up-regulated in several BC tissue samples was previously reported as up-regulated (Gottardo et al., 2007; Dyrskjot et al., 2009; Tao et al., 2011) and down-regulated (Catto et al., 2009; Wiklund et al., 2011) in BC. For the two differentially expressed markers, we calculated the AUC (area-under-the-curve) scores according to the formula AUC = (Sp + Sn)/2, where Sp is the specificity, and Sn is the sensitivity of the marker. Markers are typically considered valuable when their AUC scores exceed 0.7 (Pencina et al., 2008). According to the MA test, the AUC values for miR21 and miR205, if considered as the “BC up-regulated” markers, were 0.33 and 0.11, respectively. Therefore, according to our MA test, miR205 and miR21 showed a poor diagnostic value. Overall, we observed a large discordance between the expected (published) results and the results obtained (Figure 1A). However, due to chip limitations in this MA assay, we were not able to investigate 69 additional (miRNAs) (online Dataset 2, http://small.mrna.ru/files/).

### Experimental validation of miRNA markers with high-throughput miRNA sequencing

In a (DS) assay, we compared the sequenced miRNA fractions from nine human BC samples and from one pooled sample of six normal human bladder tissue specimens. Short RNAs (19–25 nucleotides long) were isolated and then subjected to sequencing. The number of reads varied from 1.1 to 9.6 million reads per library, with a median value of 5.1 (Supplementary Table S3). The reads were clustered using Galaxy (http://main.g2.bx.psu.edu/) and attributed to the known micro RNAs using the miRBase14.0 microRNA sequence database (Griffiths-Jones et al., 2006) and the PostParser sequence mapping tool (Baskaev et al., 2012). The number of reads that could be attributed to the known miRNAs varied between 78 and 93% in different libraries, with a median value of 87% (online Dataset 3, http://small.mrna.ru/files/).

Of the 102 published BC-associated marker miRNAs, we found 38 (37%) among the mapped and sequenced miRNAs. The expression level ratios of miRNAs in the samples under comparison (BC vs. non-BC) were evaluated by calculating the R^BC^/R^N^ ratio, where R^BC^ is a normalized number of reads in an individual miRNA molecule in a BC library, and R^N^ is a normalized number of reads of the same miRNA molecule in a mixed normal bladder tissue sample (Supplementary Table S4).

The DS data on differential expression was significantly closer to the published literature than the results received with SSH and MA methods. For each sample, we calculated the R^BC^/R^N^ ratio and then obtained the proportion of up- or down-regulated miRNAs in BC samples. MiRNA was considered to be up-regulated if the number of samples with the value R^BC^/R^N^ >2 was more than the number of samples with the value R^BC^/R^N^< 0.5. According to this criterion, 87% (33/38) of the sequenced marker miRNAs showed an expression status similar to that in the published data (Figures 1A,B). For stricter criterion values (a R^BC^/R^N^ ratio greater than 3 or less than 0.33, and greater than 4 or less than 0.25), the proportion of sequenced miRNAs in agreement with the published data was 74% (28/38) and 66% (25/38), respectively. Among the 38 investigated miRNAs that matched the DS data, 20 were up-regulated and 15 were down-regulated in BC. Three remaining miRNAs were up- and down-regulated in equal numbers to the investigated BC tissues. Six of the 38 sequenced marker miRNAs exhibited expression profiles that differed from the published data. MiR126, miR146a, miR34a, and miR493 were published as down-regulated (Lodygin et al., 2008; Saito et al., 2009; Veerla et al., 2009; Ueno et al., 2012), and miR199b and miR26b were published as up-regulated in BC (Gottardo et al., 2007; Veerla et al., 2009). However, in our experiments, we observed an opposite expression trend (Supplementary Table S4).

We calculated sensitivity scores for all of the published BC miRNA expression markers. Only four miRNAs (miR100, miR125B, miR143, and Let7c) showed the Sn values >0.7 for the strict criterion (R^BC^/R^N^ ratio > 3 or < 0.33). Two additional miRNAs (miR199A2 and miR205) showed the Sn value >0.7 only with a soft cut-off criterion R^BC^/R^N^ > 2 or < 0.5.

Importantly, a large proportion (64/102) of the published miRNA expression biomarkers was not present in our DS datasets, which may point to the absence of their expression in the investigated bladder tissue samples.

### Comparison of data on microRNA marker expression by SSH, MH, and DS analyses

Here, we aimed to evaluate the complete pool of published miRNA BC markers in a single study using three independent experimental approaches. We examined 38 of 102 published BC microRNA markers using a DS analysis of miRNA libraries, 33 miRNAs using an MH analysis, and 42 miRNAs using an SSH assay. Only our DS analysis data corresponded well with the published information. The concordance of DS results and the published data was 87%, whereas for SSH and MH results, it was 9.5 and 6%, respectively (Figures 1A–C). We were able to examine 30 published miRNA marker molecules using all three methods, and we observed only one case when all three methods agreed: miR205 was up-regulated according to all three datasets (Figure 1D; online Dataset 4, http://small.mrna.ru/files/). The congruencies between the experimental datasets are shown on Figure 1 and Supplementary Tables S5–S7 and online Datasets 5–8 (http://small.mrna.ru/files/).

### Analysis of the expression status of methylation microRNA marker genes

Using experimental assays, we analyzed two miRNA markers differentially methylated in BC according to the published data: miR744 (hypomethylated) and miR34a (hypermethylated) (Supplementary Table S8). Mir744 participates in post-transcriptional regulation of TGF-beta1 (Martin et al., 2011) which directs cellular processes such as proliferation, differentiation, migration, and survival (Blobe et al., 2000). TGF-b1 is a key regulator of embryogenesis, angiogenesis, wound healing, and inflammation. Aberrant TGF-b1 expression is implicated in carcinogenesis (Gordon and Blobe, 2008).

MiR34a directly depends on the activity of TP53 gene products (Chen and Hu, 2012). Inactivating mutations of p53, the expression of key inhibitors of p53, or genomic mutation at the p53-binding site within the miR34a gene may cause the loss of expression of miR34a. MiR34a regulates a plethora of target proteins which induce cell apoptosis; thus, it acts as a key cancer suppressor (Chen and Hu, 2012).

According to DS analysis, miR744 was up-regulated in 56% of the cases, but Mir34a was not differentially expressed in 78% of the cases and up-regulated in only 22% of the cases.

### qRT-PCR validation of data

In order to clarify what method provides the most adequate results, we measured expression of seven micro RNAs using TaqMan probe-based qRT-PCR assay. We chose one micro RNA (miR183) for which transcription was reported to be upregulated in BC, four micro RNAs (miR100, 200A, 203, 205), for which contradictory data were reported, and two micro RNAs (miR143, 199A) published as downregulated in BC. The same tissue samples as used for DS experiments were used. The results of qRT-PCR tests showed little congruence with the MH and SSH data, but were in good agreement with the DS data (Supplementary Table S9). Micro RNA expression status (up/downregulated or neutral) coincided for ~90% of the cases, varying from ~78 to 100% depending on the individual micro RNA molecule (Supplementary Table S9). We conclude, therefore, that among the three methods tested, the DS results fit best with qRT-PCR data and, thus, can be considered as the most adequate instrument to estimate miRNA content in human bladder tissues.

### Implications of differentially expressed miRNA

Previously, at least two major signaling pathways associated with BC progression were reported. Abnormal activation of the fibroblast growth factor receptor 3 gene (FGFR3) by means of either overexpression or mutation is cited in approximately 80% of non-invasive bladder tumors (Billerey et al., 2001). FGFR3 and many other growth factor receptors participate in the activation of the RAS-kinase signaling pathway, thus leading to increased cell proliferation, motility, and cancer transformation through hyperplasia of normal urothelium (Knowles, 2006).

The second major pathway associated with BC progression (P53 pathway) is misregulated in muscle-invasive tumors that mainly contain mutations in the TP53 gene (Neuzillet et al., 2012). Aberrations of the p53-induced signaling pathway lead to the development of carcinoma *in situ*, invasive carcinoma, and metastases through urothelial dysplasia (Knowles, 2006).

**Figure 2:**
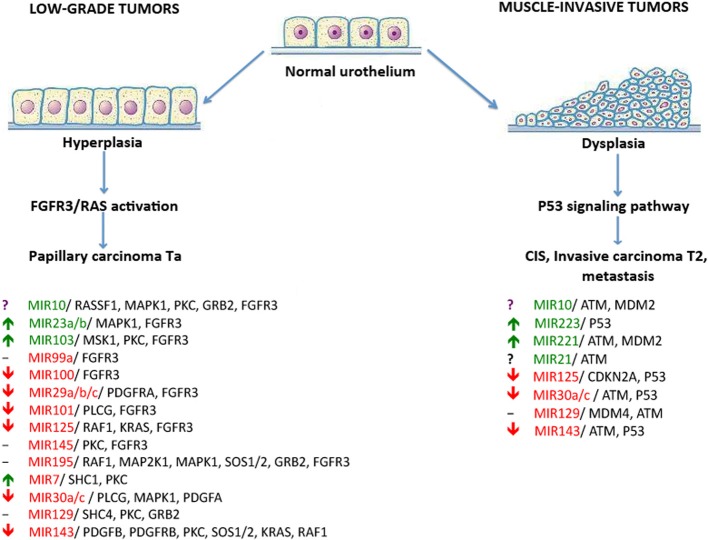
**The dual-pathway model of different types of bladder cancer development and the miRNAs that regulate these pathways**. miRNAs that were reported to be down-regulated in BC are highlighted in red; up-regulated miRNAs are highlighted in green. Arrows show up- or down-regulation of miRNAs under investigation according to DS analysis. Controversial results are marked by a “?” symbol. Unexplored miRNAs are marked by a “-” symbol.

Several studies attempted to identify miRNA molecules targeting key participants of FGFR3 and p53 signaling pathways in BC (Figure 2). Using bioinformatic methods, the Catto laboratory predicted that some miRNA molecules aberrantly expressed in BC actually targeted the FGFR3 gene product. Examples of this include miR145, miR101, miR99a, and miR100. Furthermore, the regulation of FGFR3 by miR99a and miR100 was experimentally validated (Catto et al., 2009). An increase in miR143 expression in BC cells is accompanied by the lower expression of RAS genes. Another line of evidence recently confirmed this by showing that induced transcription of miR143 in BC cell lines led to a decreased expression of RAS (Lin et al., 2009; Kent et al., 2010). Another study showed that a decrease in miR7 expression is frequently associated with the hyperactive FGFR3 mutation status found in BC tissues. Key elements of the p53 signaling pathway—MDM2, MDM4, and ATM gene products—were predicted as targets for miR10. MiR129 potentially targets MDM4 and ATM in this pathway; miR125b, miR43, miR30a/c, and miR223 were predicted to target p53 directly (Fendler et al., 2011). In concordance with these findings, it was reported that expression levels of miR10, miR125b, and miR222 may serve as predictors of muscle-invasive carcinomas (Veerla et al., 2009). Using the DS approach, we assayed 12 of 18 published BC marker miRNAs that were predicted to target either FGFR3 or TP53 pathways. Our experimental data matched the published data for seven miRNAs (miRNAs 29, 30, 100, 101, 125, and 143 were down-regulated in BC and miR103 was up-regulated) (Figure 2). For miR100 and miR143, we observed a total suppression of expression in all studied cases. For miR21 and miR10, we obtained contradictory data: different patients showed either up- or down-regulation of their expression. Using DS analysis, we confirmed the expression of 70% of the published miRNAs which were predicted to be participants in BC-associated signaling pathways (Figure 2).

## Discussion

MiRNAs can be used as biomarkers for many types of cancers (Van Roosbroeck et al., 2013). Some miRNAs may help trace the tissue of origin of cancers whose primary origin is unknown (Rosenfeld et al., 2008). In addition, miRNA molecules are advantageous for molecular diagnostics due to their greater stability *in vitro* compared to mRNA molecules (Jung et al., 2010).

In this study, we used different methods to profile miRNAs for the evaluation of the potential utility of published miRNA markers for BC diagnostics. We used (MH), subtractive suppression hybridization (SSH), and (DS) technologies to determine miRNA expression profiles in 17 tissue samples of urothelial bladder carcinoma and in eight histologically normal urothelial samples.

Among the 95 published miRNA expression markers, only 43 were detected in our samples using the SSH approach followed by DS; 34 markers were detected using MH analysis; 38 were detected using the DS assay.

The higher expression level of miR205 in cancer was confirmed by all experimental assays. It was previously shown that ectopic expression of miRNA205 induces apoptosis, cell cycle arrest, impaired cell viability, cloning, and invasive properties of cancer cells (Yue et al., 2012). MiR205 can specifically suppress VEGF-A expression by directly interacting with the putative miRNA-205 binding site at the 3′-UTR (Yue et al., 2012). MiR205 also regulates the expression of tumor-suppressor, PTEN. The introduction of miR205 into CNE-2 cells suppresses PTEN protein expression followed by the activation of AKT, an increased number of foci formation, and the reduction of post-irradiation cell apoptosis (Qu et al., 2012). MiR205 was also reported to be aberrantly expressed in breast (Adachi et al., 2011), prostate (Bhatnagar et al., 2010), lung (Tellez et al., 2011), head, and neck (Kimura et al., 2010), and other cancers.

MA analysis and SSH showed poor correlation between miRNA expression and the published data, with the largest number of identified miRNAs characterized as “intact” (not differentially expressed). This may correspond to the methodological issues inherent in the preparation of cDNA library for both assays. Oligo-dT primers were used to initiate the synthesis of first-strand cDNAs for MA and SSH assays. However, most mature miRNAs may lack poly (A) sequences at their 3′ termini and thus escape such types of analysis. Nevertheless, low convergence of MA and SSH results (without DS analysis) with the qRT-PCR data and with the published information suggest that they are not informative for studying miRNA expression profiles. Theoretically, variations of the MA- and of the SSH-based techniques that do not rely on the amplification of poly(A)+ sequences may show somewhat better results for miRNA profiling, but this will be a matter of further studies.

In contrast, the development of high-throughput DS technology provides an opportunity for almost complete analyses of miRNA profiles. DS reveals an abundance of miRNAs and can identify miRNAs missed by traditional cloning and sequencing methods (Hurd and Nelson, 2009). At present, DS is considered to be the gold standard for high-throughput analysis of miRNAs (Han et al., 2011). Our qRT-PCR experiments confirmed that among the tested techniques, DS should be used as the method of choice for assessing miRNA expression in human bladder.

To our knowledge, this is the first systematic study evaluating the diagnostic potential of published miRNA biomarkers for BC by large-scale profiling of miRNA expression in pathological human tissue samples. Our findings suggest that DS analysis is most likely the best approach for the identification and evaluation of the diagnostic and/or prognostic value of miRNA cancer biomarkers.

Despite the obvious significance of miRNAs for tumor progression, the relatively sparse data on miRNAs associated with bladder oncology suggests that this area necessitates future research.

### Conflict of interest statement

The authors declare that the research was conducted in the absence of any commercial or financial relationships that could be construed as a potential conflict of interest.
