# Supplementary material for: A systematic experimental evaluation of microRNA markers of human bladder cancer
Source: Front Genet. 2013 Nov 15;4:247. doi: 10.3389/fgene.2013.00247 (PMC3828615; doi:10.3389/fgene.2013.00247)
Supplement: Supplementary file 1 [file DataSheet1.ZIP › 61677_Buzdin_Data_Sheet_2.DOCX]

**Supplementary Table 2. SSH method description and oligonucleotides used in SSH experiments.**

***cDNA library generation for SSH and subtraction procedure***

SMART Oligo II oligonucleotide and CDS primer were used for first-strand cDNA synthesis. First-strand cDNA synthesis was started from 0·5 ug RNA in a total reaction volume of 10 µl. One µl of 5-times diluted first-strand cDNA was then used for PCR amplification with SMART PCR primer. Eighteen PCR cycles for sample #1 and 19 PCR cycles for sample #2 were performed. Each cycle included 95^o^C for 10 s; 65.5^o^C for 20 s; 72^o^C for 3 min. SMART-amplified cDNA samples were further digested by Rsa I endonuclease.

***Subtractive hybridization***

Subtractive hybridization was performed using the SSH method in both directions (#1 vs #2 and #2 vs #1) as described [16]. Briefly, the following procedures were performed. For each direction, two tester populations were created by ligating different suppression adapters (Adapters 1 and 2R). Tester populations were then mixed with 30X driver excess (driver cDNA had no adaptors) in two separate tubes, denatured, and allowed to renature. After first hybridization, the two samples were mixed and hybridized together. Subtracted cDNA was then amplified by primary and secondary PCR. For primary PCR, 26 PCR cycles with PCR primer 1 were performed for subtracted 1 cDNA and 26 cycles for subtracted 2 cDNA. Secondary (nested) PCR included 10 PCR cycles with nested primers 1and 2R were performed for both subtracted cDNA samples.

| SMART Oligo II A  oligonucleotide | 5’-AAGCAGTGGTATCAACGCAGAGTACGCrGrGrG-3’ |
| --- | --- |
| SMART CDS  primer II A | 5’-AAGCAGTGGTATCAACGCAGAGTA-d(T)30-3’ |
| SMART PCR  primer II A | 5’-AAGCAGTGGTATCAACGCAGAGT-3’ |
| Adapter 1 | 5’-CTAATACGACTCACTATAGGGCTCGAGCGGCCGCCCGGGCAGGT-3’  3’-GGCCCGTCCA-5’ |
| PCR primer 1 | 5'–CTAATACGACTCACTATAGGGC-3' |
| Nested primer 1 | 5'–TCGAGCGGCCGCCCGGGCAGGT–3' |
| Adapter 2R | 5'–CTAATACGACTCACTATAGGGCAGCGTGGTCGCGGCCGAGGT–3'  3'–GCCGGCTCCA–5' |
| Nested primer 2R | 5'–AGCGTGGTCGCGGCCGAGGT–3' |
