# Supplementary material for: A systematic experimental evaluation of microRNA markers of human bladder cancer
Source: Front Genet. 2013 Nov 15;4:247. doi: 10.3389/fgene.2013.00247 (PMC3828615; doi:10.3389/fgene.2013.00247)
Supplement: Supplementary file 1 [file DataSheet1.ZIP › 61677_Buzdin_Data_Sheet_1.DOCX]

Supplementary Table 1. Patient information and clinical material characteristics

| **Number** | **Sex (M – male, F – female)** | **Age, years** | **Tumor TNM classification and grade (G)** | **Primary or recurrent tumor growth** |
| --- | --- | --- | --- | --- |
| **2** | M | 60 | T1N0M0, G3 | Recurrence |
| **3** | F | 77 | T2N0M0, G2 | Primary |
| **4** | M | 62 | T4aNxM0, G3 | Recurrence |
| **5** | M | 58 | T4aN1M0, G3 | Primary |
| **6** | M | 57 | T3aN0M0, G2 | Recurrence |
| **7** | M | 59 | T1NxM0, G3 | Recurrence |
| **8** | M | 48 | T4aN2M0, G3 | Recurrence |
| **55** | M | 55 | T1N0M0, G3 | Primary |
| **56** | F | 66 | T3bN0M0, G3 | Primary |
| **58** | M | 75 | T1NxM0, G3 | Recurrence |
| **60** | M | 71 | T1NxM0, G1 | Primary |
| **61** | M | 63 | T3bN0M0, G2 | Recurrence |
| **64** | M | 82 | T1NxM0, G1 | Recurrence |
| **66** | M | 56 | T3bN1M0, G2 | Recurrence |
| **68** | M | 62 | T4N0M0, G1 | Primary |
| **69** | M | 64 | T1N0M0, G1 | Primary |
| **72** | M | 44 | T1N0M0, G2 | Primary |
