# Supplementary material for: A systematic experimental evaluation of microRNA markers of human bladder cancer
Source: Front Genet. 2013 Nov 15;4:247. doi: 10.3389/fgene.2013.00247 (PMC3828615; doi:10.3389/fgene.2013.00247)
Supplement: Supplementary file 1 [file DataSheet1.ZIP › 61677_Buzdin_Data_Sheet_10.DOC]

**Supplementary Table 3. Characterization of sequenced small RNA libraries**

Sample ID, a name of an individual bladder cancer clinical sample.

Counts, the number of individual sequences in the library; cut-off, ten copies per individual sequence per library.

Reads, the total number of reads obtained for individual samples.

**Supplementary Table 4. Comparison of deep sequencing data with the published data on bladder cancer-linked micro RNAs**

The published data, the micro RNA status according to the previously published data. “UP” means the upregulated status, “DOWN” – the downregulated status, “UP OR DOWN” – contradictory data reported.

Deep sequencing results, the experimentally obtained cancer-to-norm ratio for every BC tissue sample calculated according to the formula R_BC_/R_Norm,_ where R_BC_ is the normalized number of reads in an individual BC sample, and R_Norm_ is the normalized number of reads in a sample of pooled normal bladder tissues. The red color means down-regulated expression compared to pooled normal tissues, the green color – up-regulated expression and the white color – the intact status (the cut-off rate is 2-fold change in the normalized number of reads). “ND” means no sequence data.

Sn value 1, the sensitivity calculated according to differential expression criteria corresponding to the 2-fold change cut-off rate in the normalized number of reads.

Sn value 2, the sensitivity calculated according to differential expression criteria corresponding to the 3-fold change cut-off rate in the normalized number of reads.

Sn value 3, the sensitivity calculated according to differential expression criteria corresponding to the 4-fold change cut-off rate in the normalized number of reads.

**Supplementary Table 5. Comparison of SSH and microarray data with the published data on bladder cancer-linked micro RNAs**

The published data, the micro RNA status according to the previously published data. “UP” means the up-regulated status, “DOWN” – the down-regulated status, “UP OR DOWN” – contradictory data reported.

SSH results, the experimentally obtained cancer-to-norm ratio calculated according to the formula R_BC_/R_Norm,_ where R_BC_ is the normalized number of reads in a mixed BC sample, and R_Norm_ is the normalized number of reads in a mixed sample of normal bladder tissues. The red color means down-regulated expression compared to pooled normal tissues, the green color – up-regulated expression, and the white color – the intact status (the cut-off rate is 2-fold change in the normalized number of reads).

Microarray results, the experimentally obtained cancer-to-norm ratio calculated according to the formula F_BC_/F_Norm,_ where F_BC_ is a normalized fluorescent signal in an individual BC sample and F_Norm_ is the mean normalized fluorescence in samples of normal bladder tissues. The red color (no incidences) means down-regulated expression compared to pooled normal tissues, the green color – up-regulated expression, and the white color – the intact status (the cut-off rate is 2-fold change in the normalized fluorescence).

**Supplementary Table 6. Comparison of SSH and deep sequencing data with the published data on bladder cancer-linked micro RNAs**

The published data, the micro RNA status according to the previously published data. “UP” means the up-regulated status, “DOWN” – the down-regulated status, “UP OR DOWN” – contradictory data reported.

SSH results, the experimentally obtained cancer-to-norm ratio calculated according to the formula R_BC_/R_Norm,_ where R_BC_ is the normalized number of reads in a mixed BC sample and R_Norm_ is the normalized number of reads in a mixed sample of normal bladder tissues. The red color means down-regulated expression compared to pooled normal tissues, the green color – up-regulated expression, and the white color – the intact status (the cut-off rate is 2-fold change in the normalized number of reads).

Deep sequencing results, the experimentally obtained cancer-to-norm ratio for every BC tissue sample calculated according to the formula R_BC_/R_Norm,_ where R_BC_ is the normalized number of reads in an individual BC sample and R_Norm_ is the normalized number of reads in a sample of pooled normal bladder tissues. The red color means down-regulated expression compared to pooled normal tissues, the green color – up-regulated expression, and the white color – the intact status (the cut-off rate is 2-fold change in the normalized number of reads). “ND” means no sequence data.

**Supplementary Table 7. Comparison of microarray and deep sequencing data with the published data on bladder cancer-linked micro RNAs**

The published data, the micro RNA status according to the previously published data. “UP” means the up-regulated status, “DOWN” – the down-regulated status, “UP OR DOWN” – contradictory data reported.

Deep sequencing results, the experimentally obtained cancer-to-norm ratio for every BC tissue sample calculated according to the formula R_BC_/R_Norm,_ where R_BC_ is the normalized number of reads in an individual BC sample and R_Norm_ is the normalized number of reads in a sample of pooled normal bladder tissues. Thered color means down-regulated expression compared to pooled normal tissues, the green color – up-regulated expression and the white color – the intact status (the cut-off rate is 2-fold change in the normalized number of reads). “ND” means no sequence data.

Microarray results, the experimentally obtained cancer-to-norm ratio calculated according to the formula F_BC_/F_Norm,_ where F_BC_ is the normalized fluorescent signal in an individual BC sample and F_Norm_ is the mean normalized fluorescence in a samples of normal bladder tissues. The red color (no incidences) means down-regulated expression compared to pooled normal tissues, the green color – up-regulated expression, and the white color – the intact status (the cut-off rate is 2-fold change in the normalized fluorescence).

**Supplementary Table 8. Comparison of deep sequencing, microarray and SSH datasets with the published data on bladder cancer-linked micro RNAs**

MIR marker name, according to UCSC genome browser (<http://genome.ucsc.edu/>)

MIRbase name, micro RNA names according to MIRbase database nomenclature (http://www.mirbase.org/)

The published data, the micro RNA status according to the previously published data. “UP” means the up-regulated status, “DOWN” – the down-regulated status, “UP OR DOWN” – contradictory data reported.

PMID, PubMed ID for references reporting the role of micro RNA in bladder cancer.

Sequence, the complete 5’-3’ sequence of microRNA.

Length, micro RNA length in bases.

Reads number/ P55, etc. – the normalized number of reads for an individual sample in deep sequencing experiments.

DS/P55, etc. – the ratio of the normalized number of reads in an individual bladder cancer sample to the normalized number of reads in a mixed normal tissue sample.

MH/P55, etc. – the experimentally obtained microarray cancer-to-norm ratio calculated according to the formula F_BC_/F_Norm,_ where F_BC_ is the normalized fluorescent signal in an individual BC sample and F_Norm_ is the mean normalized fluorescence in a samples of normal bladder tissues. The red color (no incidences) means down-regulated expression compared to pooled normal tissues, the green color – up-regulated expression, and the white color – the intact status (the cut-off rate is 2-fold change in the normalized fluorescence).

SSH results, the experimentally obtained cancer-to-norm ratio calculated according to the formula R_BC_/R_Norm,_ where R_BC_ is the normalized number of reads in a mixed BC sample and R_Norm_ is the normalized number of reads in a mixed sample of normal bladder tissues. The red color means down-regulated expression compared to pooled normal tissues, the green color – up-regulated expression, and the white color – the intact status (the cut-off rate is 2-fold change in the normalized number of reads).

**Supplementary Table 9. Comparison of deep sequencing and qRT-PCR datasets with the published data on bladder cancer-linked micro RNAs**

MIR marker name, according to UCSC genome browser (<http://genome.ucsc.edu/>)

The published data, the micro RNA status according to the previously published data. “UP” means the up-regulated status, “DOWN” – the down-regulated status, “UP OR DOWN” – contradictory data reported.

Deep sequencing results, P55, etc. – the ratio of the normalized number of reads in an individual bladder cancer sample to the normalized number of reads in a mixed normal tissue sample.

qRT-PCR data, P55, etc. – the ratio of the normalized miRNA concentration in an individual bladder cancer sample to the normalized miRNA concentration in a mixed normal tissue sample.
